# Grain Growth Behavior and Electrical Properties of 0.96(K_0.46−x_Na_0.54−x_)Nb_0.95_Sb_0.05_O_3_–0.04Bi_0.5_(Na_0.82_K_0.18_)_0.5_ZrO_3_ Ceramics

**DOI:** 10.3390/ma15072357

**Published:** 2022-03-22

**Authors:** Yeon-Ju Park, Il-Ryeol Yoo, Seong-Hui Choi, Jiung Cho, Kyung-Hoon Cho

**Affiliations:** 1 School of Materials Science and Engineering, Kumoh National Institute of Technology, Gumi 39177, Korea; pyjuuu0902@kumoh.ac.kr (Y.-J.P.); yiy1204@kumoh.ac.kr (I.-R.Y.); shchoi7@kumoh.ac.kr (S.-H.C.); 2 Western Seoul Center, Korea Basic Science Institute, Seoul 03579, Korea

**Keywords:** abnormal grain growth, lead-free, piezoelectric, 2D nucleation, vacancy

## Abstract

This study investigated the causes of microstructural changes and the resultant electrical properties according to the sintering temperature of 0.96(K_0.46−x_Na_0.54−x_)Nb_0.95_Sb_0.05_O_3_-0.04Bi_0.5_(Na_0.82_K_0.18_)_0.5_ZrO_3_ lead-free ceramics by analyzing the correlation between vacancy concentrations and 2D nucleation. When sintered for 4 h, no grain growth occurred for the x = 0.000 composition over a wide temperature range, demonstrating that the existence of initial vacancies is essential for grain growth. As x increased, that is, as the vacancy concentration increased, the critical driving force (ΔG_C_) for 2D nucleation decreased, and abnormal grain growth was promoted. The number and size of these abnormal grains increased as the sintering temperature increased, but at sintering temperatures above 1100 °C, they decreased again owing to a large drop in ΔG_C_. The x = 0.005 specimen sintered at 1085 °C exhibited excellent piezoelectric properties of d_33_ = 498 pC/N and k_p_ = 0.45 due to the large number of large abnormal grains with an 83% tetragonal phase fraction. The x = 0.000 specimen sintered at 1130 °C with suppressed grain growth exhibited good energy storage properties because of its very high relative density and small grain size of 300 to 400 nm.

## 1. Introduction

Piezoelectric ceramics are smart materials that enable the interconversion between mechanical and electrical energy and are used in various electronic devices such as actuators, transducers, sensors, and motors [1,2,3,4,5]. Most piezoelectric ceramic materials have been commercialized, mainly for Pb(Zr,Ti)O_3_ (PZT)-based ceramics with a high lead content. However, recently tightened environmental regulations around the world have urged the development of lead-free piezoelectric ceramics that do not contain lead oxide, which leads to harmful effects such as high toxicity and high vapor pressure in manufacturing and disposal processes [2,6,7,8]. (K,Na)NbO_3_ (KNN)-based ceramics are ferroelectric materials with ABO_3_–type perovskite structures and are actively being studied as eco-friendly lead-free piezoelectric materials because of their high Curie temperatures and good piezoelectric properties [6,7,8,9,10]. High piezoelectric coefficient values can be obtained compared to pure KNN by modifying KNN with various elements (A-site: Li, Bi, etc., B-site: Ta, Sb, etc.) to move orthorhombic-tetragonal (O-T) and rhombohedral-orthorhombic (R-O) phase transition temperatures (T_O-T_ and T_R-O_) to near room temperature [11,12,13,14]. In addition, when T_O-T_ and T_R-O_ meet near room temperature by doping Li, Bi, Ag, etc. at the A-site and Sb, Zr, Hf, etc. in the B-site of KNN, excellent piezoelectric properties comparable to those of PZT-based ceramics are obtained because of the coexistence of rhombohedral and tetragonal phases [15,16,17,18,19,20,21,22,23].

For the commercialization of KNN-based ceramics, not only excellent piezoelectric properties but also reproducibility of microstructures directly related to the piezoelectric properties are very important. The microstructure of KNN-based ceramics can be sensitively changed depending on the sintering conditions, and various microstructures and piezoelectric properties with large deviations have been reported, even in materials with the same composition [24,25,26,27]. This is a major obstacle in ensuring the reproducibility of piezoelectric characteristics. 

The grains of KNN-based ceramics exhibit a faceted cube shape, signifying their very high interfacial energy anisotropy [28,29,30]. The faceted grain boundary makes normal grain growth difficult, owing to the absence of kink sites for the adsorption of atoms, which is associated with the low sinterability of KNN-based ceramics. The grain growth of ceramics with faceted grain boundaries can be enabled by 2D nucleation and growth mechanisms [28,29,31]. The critical driving force (ΔG_C_) for the formation of 2D nuclei on the grain surface is expressed as

(1) $$\Delta G_{C}=\frac{{\Omega \varepsilon }^{2}}{3\mathrm{hkT}}\;,$$

where ε is the step free energy of the 2D nucleus, Ω is the molar volume, k is Boltzmann’s constant, T is the temperature, and h is the height of the 2D nucleus. The driving force (ΔG) for grain growth of the grains is given as follows:

(2) $$\Delta G=2\gamma_{\mathrm{sl}}\Omega \left(\frac{1}{r^{*}}-\frac{1}{r}\right),$$

where γ_sl_ is the solid-liquid interfacial energy, r is the radius of the growing grain, and r^∗^ is the average radius of the grains around the growing grain. Only grains that satisfy the condition ΔG ≥ ΔG_C_ can grow via 2D nucleation. The variable ε in Equation (1) is inversely proportional to the configurational entropy; therefore, it can be decreased as the vacancy increases [29,30].

In this study, 0.96(K_0.46−x_Na_0.54−x_)Nb_0.95_Sb_0.05_O_3_-0.04Bi_0.5_(Na_0.82_K_0.18_)_0.5_ZrO_3_ compositions with intentionally reduced alkali element content were designed, and the effect of vacancy concentration on the microstructure and electrical properties of sintered ceramics was investigated. It was found that grain growth was suppressed in compositions in which the alkali element content was not controlled, and there were specific compositions and sintering temperatures to obtain a microstructure exhibiting excellent piezoelectric properties. In addition, we report that when a highly dense microstructure with suppressed grain growth is secured, the energy storage performance is enhanced, even if the ferroelectricity is weakened owing to the small grain size.

## 2. Experimental

**Scheme 1:**
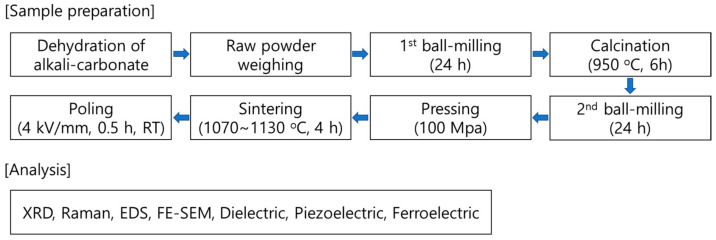
Schematic diagram of the experimental procedure in this study.

Scheme 1 shows the experimental procedure including sample preparation and analysis methods.

The 0.96(K_0.46−x_Na_0.54−x_)Nb_0.95_Sb_0.05_O_3_-0.04Bi_0.5_(Na_0.82_K_0.18_)_0.5_ZrO_3_ compositions (x = 0.000, 0.005, 0.010, 0.015) were prepared using K_2_CO_3_ (99% purity), Na_2_CO_3_ (99% purity), Nb_2_O_5_ (99.9% purity), Sb_2_O_3_ (99.9% purity), Bi_2_O_3_ (99.9% purity), and ZrO_2_ (98% purity) raw powders (all from Kojundo Korea Co., Uiwang-si, Korea) by solid state synthesis. K_2_CO_3_ and Na_2_CO_3_ powders were heated at 400 °C for 24 h under a vacuum to achieve complete dehydration. The raw powders were mixed according to their compositions and milled with ethanol and yttria-stabilized zirconia balls in a polyethylene jar for 24 h. The powder mixtures were calcined at 950 °C for 6 h with a heating rate of 4 °C/min and naturally cooled down to room temperature in a furnace (furnace-cooling). The calcined powders were re-milled for 24 h and pressed into disk-shaped powder compacts at 100 MPa. The powder compacts were sintered for 4 h at temperatures ranging from 1070 °C to 1130 °C with a heating rate of 4 °C/min and naturally cooled down to room temperature in a furnace.

An Ag paste was printed on both surfaces of the sintered specimens and fired at 600 °C for 30 min with a heating rate of 4 °C/min and naturally cooled down to room temperature in a furnace. The specimens were electrically poled by applying a DC electric field of 4 kV/mm for 30 min at room temperature in a silicone oil bath. Structural analyses were performed using X-ray diffraction (XRD, D-MAX/2500, Rigaku, Japan), Raman spectroscopy (System 1000, Renishaw, UK), energy-dispersive X-ray spectroscopy (EDS; XFlash 630, Bruker Nano GmbH, Berlin, Germany), and scanning electron microscopy (SEM, JSM-6500F, JEOL, Akishima, Japan). The piezoelectric and dielectric properties of the sintered specimens were measured using a d_33_-m (YE2730A, Sinocera Piezotronics, Yangzhou, China) and impedance analyzer (IM3570, Hioki, Japan). Polarization vs. electric field (P-E) curves were measured using a ferroelectric test system (PK-CPE1801, PolyK Technologies, Philipsburg, PA, USA).

## 3. Results and Discussion

**Figure 1:**
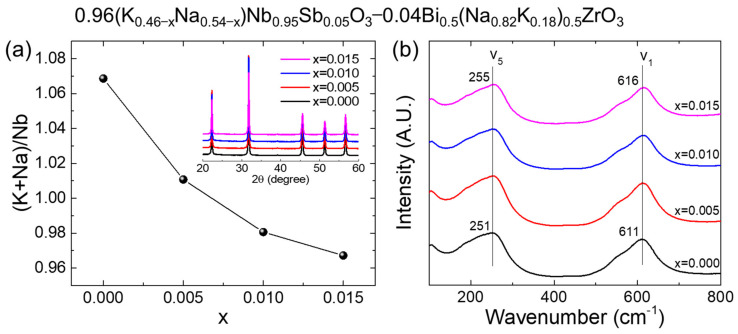
(**a**) Ratio of alkali-ion content to niobium ion content of 0.96(K_0.46−x_Na_0.54−x_)Nb_0.95_Sb_0.05_O_3_-0.04Bi_0.5_(Na_0.82_K_0.18_)_0.5_ZrO_3_ calcined powders obtained by EDS analysis. Inset shows their XRD patterns. (**b**) Raman spectra of 0.96(K_0.46−x_Na_0.54−x_)Nb_0.95_Sb_0.05_O_3_-0.04Bi_0.5_(Na_0.82_K_0.18_)_0.5_ZrO_3_ calcined powders.

Figure 1a shows the ratio of alkali ion content to Nb ion content in 0.96(K_0.46−x_Na_0.54−x_)Nb_0.95_Sb_0.05_O_3_-0.04Bi_0.5_(Na_0.82_K_0.18_)_0.5_ZrO_3_ powders calcined at 950 °C, and the ratio decreased as x increased. As all of the compositions exhibit perovskite structures without a secondary phase (inset in Figure 1a), the concentration of alkali ion vacancies is expected to increase as x increases. As shown in Figure 1b, the v_1_ and v_5_ mode peaks of the Raman spectra shifted toward higher wavenumbers as x increased.

The v_1_ and v_5_ modes correspond to the stretching and bending modes of the B-site octahedra (NbO_6_), respectively, and the v_1_ peak shifts to a higher wavenumber when an alkali ion deficiency (or vacancy) exists or when ions with a small ionic radius, such as lithium, are substituted for the A-site [32,33,34]. This also demonstrates that the concentration of alkali ion vacancies present in the 0.96(K_0.46−x_Na_0.54−x_)Nb_0.95_Sb_0.05_O_3_-0.04Bi_0.5_(Na_0.82_K_0.18_)_0.5_ZrO_3_ grains increases with an increase in x.

**Figure 2:**
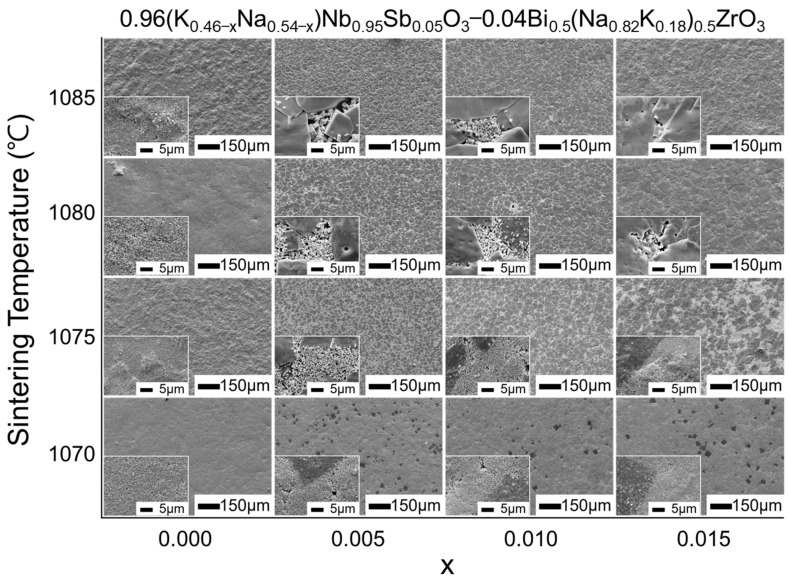
SEM images showing the microstructural evolution of 0.96(K_0.46−x_Na_0.54−x_)Nb_0.95_Sb_0.05_O_3_-0.04Bi_0.5_(Na_0.82_K_0.18_)_0.5_ZrO_3_ ceramics sintered in the temperature range from 1070 to 1085 °C for 4 h. Insets show magnified SEM images for each specimen.

Figure 2 shows microstructures of 0.96(K_0.46−x_Na_0.54−x_)Nb_0.95_Sb_0.05_O_3_-0.04Bi_0.5_(Na_0.82_K_0.18_)_0.5_ZrO_3_ ceramics sintered for 4 h at temperatures ranging from 1070 to 1085 °C. 

Unusually, no grain growth was observed in the x = 0 specimen under all sintering temperatures, signifying that 2D nucleation was suppressed in all grains owing to the high ΔG_C_ because the vacancy concentration of grains was too low. However, for x = 0.005, 0.010, and 0.015 specimens sintered at 1070 °C, abnormal grains of several tens of micrometers in size were observed in the small matrix grains that did not grow. This can be interpreted as 2D nucleation and growth progressing in some grains, satisfying the ΔG ≥ ΔG_C_ condition as ε decreases owing to the increased vacancy concentration. Moreover, the number and size of these abnormal grains increased as the sintering temperature increased. This implies that the higher the sintering temperature, the more active the alkali element volatilization, which increases the number of grains satisfying the ΔG ≥ ΔG_C_ condition and advances the onset of 2D nucleation during sintering. Matrix grains of the x = 0.005, 0.010, and 0.015 specimens sintered at temperatures below 1080 °C did not grow. However, the x = 0.005 specimen sintered at 1085 °C exhibited a microstructure composed of large grains of several tens of micrometers and small grains of several micrometers, suggesting that the ΔG_C_ continued to decrease with time at 1085 °C, and the growth of small-sized grains was also initiated.

**Figure 3:**
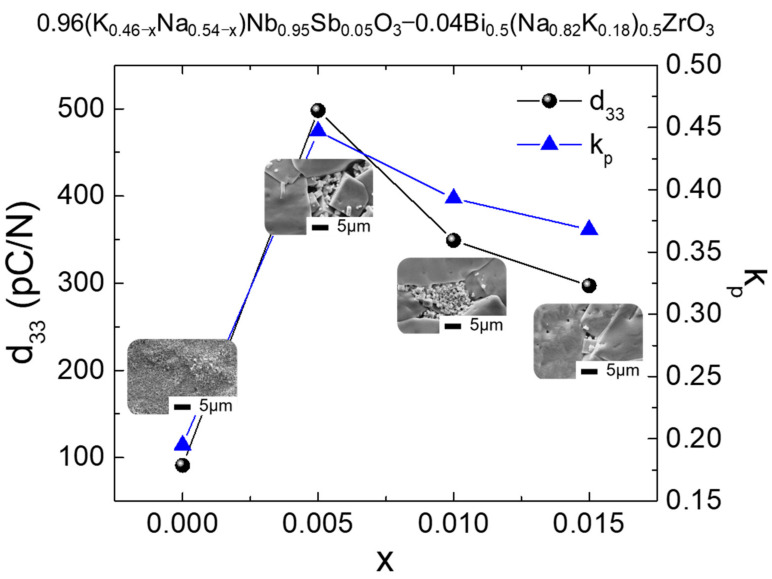
Piezoelectric charge coefficient (d_33_) and electromechanical coupling coefficient (k_p_) of 0.96(K_0.46−x_Na_0.54−x_)Nb_0.95_Sb_0.05_O_3_-0.04Bi_0.5_(Na_0.82_K_0.18_)_0.5_ZrO_3_ ceramics sintered at 1085 °C for 4 h.

Figure 3 shows the piezoelectric properties of 0.96(K_0.46−x_Na_0.54−x_)Nb_0.95_Sb_0.05_O_3_-0.04Bi_0.5_(Na_0.82_K_0.18_)_0.5_ZrO_3_ ceramics sintered at 1085 °C for 4 h. The x = 0.000 specimen, in which grain growth did not occur, exhibited poor piezoelectric properties of d_33_ < 100 pC/N and k_p_ < 0.2, whereas the x = 0.005 specimen, in which large grain growth was observed, exhibited excellent piezoelectric properties of d_33_ = 498 pC/N and k_p_ = 0.45. A large grain size makes domain rotation easier, thereby increasing the piezoelectric properties [26,35]. However, in the case of the x = 0.010 and 0.015 specimens, the piezoelectric properties were reduced compared with those of the x = 0.005 specimen, although they displayed a microstructure composed of large grains similar to that of the x = 0.005 specimen.

**Figure 4:**
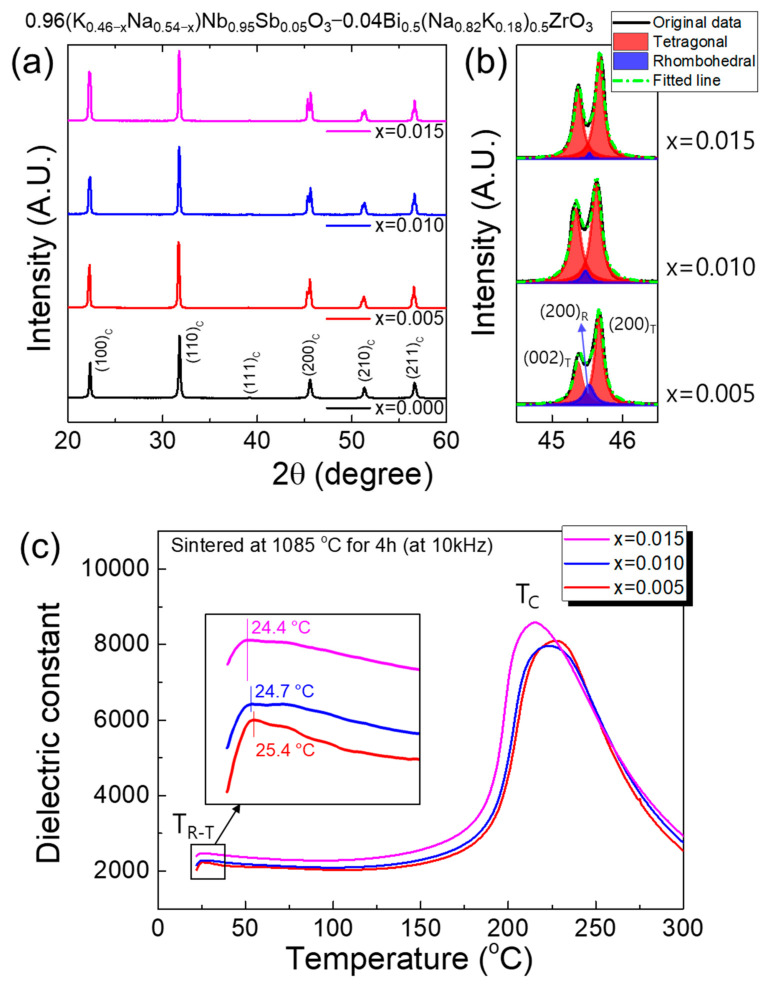
(**a**) XRD patterns of 0.96(K_0.46−x_Na_0.54−x_)Nb_0.95_Sb_0.05_O_3_-0.04Bi_0.5_(Na_0.82_K_0.18_)_0.5_ZrO_3_ ceramics sintered at 1085 °C for 4 h. (**b**) Finely scanned XRD patterns for (200)_C_ peaks of x = 0.005, 0.010, and 0.015 specimens. (**c**) Dielectric constant vs. temperature curves of x = 0.005, 0.010, and 0.015 specimens.

The 0.96(K_0.46−x_Na_0.54−x_)Nb_0.95_Sb_0.05_O_3_-0.04Bi_0.5_(Na_0.82_K_0.18_)_0.5_ZrO_3_ ceramics sintered at 1085 °C for 4 h showed a pure perovskite structure without a secondary phase, as shown in Figure 4a. The x = 0.000 specimen exhibited a pseudo-cubic structure owing to its very small grain size. However, clear separation of the (200)_C_ peak was observed for the x = 0.005, 0.010, and 0.015 specimens composed of large grains. Deconvolution analysis of the (200)_C_ peaks in Figure 4b confirmed that x = 0.005, 0.010, and 0.015 compositions were in a tetragonal-rich rhombohedral-tetragonal (R-T) phase coexistence (or phase transition) state at room temperature [36,37]. The rhombohedral and tetragonal phase fractions are shown in Table 1. It can be seen that the fraction of the rhombohedral phase decreases as x increases. Furthermore, as x increased, both the R-T transition temperature (T_R-T_) and Curie temperature (T_C_) shifted toward lower temperatures (Figure 4c), which supports the results in Table 1. The piezoelectric properties of the R-T phase coexistence compositions are maximized when the fraction of the tetragonal phase is approximately 80% [38,39]. The deterioration of the piezoelectric properties of the x = 0.010 and 0.015 specimens observed in Figure 3 might be due to the high tetragonal phase fraction of more than 90%. Therefore, even for specimens with similar microstructures, their phase status and piezoelectric properties may vary significantly depending on the initial vacancy concentration. For the compositions used in this study, an excessively high vacancy concentration seems unsuitable for securing excellent piezoelectric properties.

**Figure 5:**
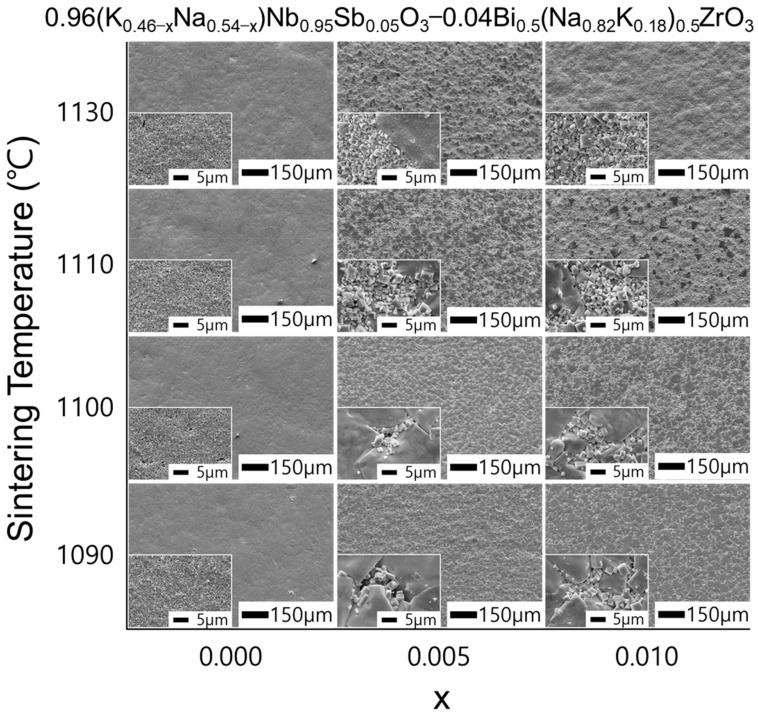
SEM images showing the microstructural evolution of 0.96(K_0.46−x_Na_0.54−x_)Nb_0.95_Sb_0.05_O_3_-0.04Bi_0.5_(Na_0.82_K_0.18_)_0.5_ZrO_3_ ceramics sintered in the temperature range from 1090 to 1130 °C for 4 h. Insets show magnified SEM images for each specimen.

Figure 5 shows the microstructures of 0.96(K_0.46−x_Na_0.54−x_)Nb_0.95_Sb_0.05_O_3_-0.04Bi_0.5_(Na_0.82_K_0.18_)_0.5_ZrO_3_ ceramics sintered at temperatures higher than 1085 °C. For the x = 0.000 specimen, grain growth was not observed even at a very high sintering temperature of 1130 °C, which indicates that the ΔG_C_ value of the stoichiometric composition, in which the content of the initial alkali element was not reduced, could be maintained at a very high level even at a high sintering temperature. In the case of the x = 0.005 specimen, as the sintering temperature increased, the number of large abnormal grains decreased, and the matrix grains grew to a size of several micrometers. This phenomenon was also observed in the x = 0.010 specimen, but the number of abnormal grains began to decrease at a lower sintering temperature (1100 °C) than that of the x = 0.005 specimen (1110 °C). The x = 0.010 specimen sintered at 1130 °C displayed a microstructure in which all grains were grown to a size of several micrometers without large abnormal grains.

The decrease in the number of abnormal grains as the sintering temperature increased is considered to be due to the increase in the vacancy concentration due to the enhanced volatilization of the alkali element. ΔG_C_ decreases as the concentration of vacancies increases, and if the number of grains satisfying the ΔG ≥ ΔG_C_ condition rapidly increases during the sintering process, the growth of large abnormal grains is inevitably suppressed, and the number of abnormal grains is also limited. In the case of the x = 0.010 specimen, because the initial vacancy concentration was higher than that of the x = 0.005 specimen, ΔG_C_ decreased more rapidly, and the abnormal grain growth started to be suppressed at lower temperatures.

**Figure 6:**
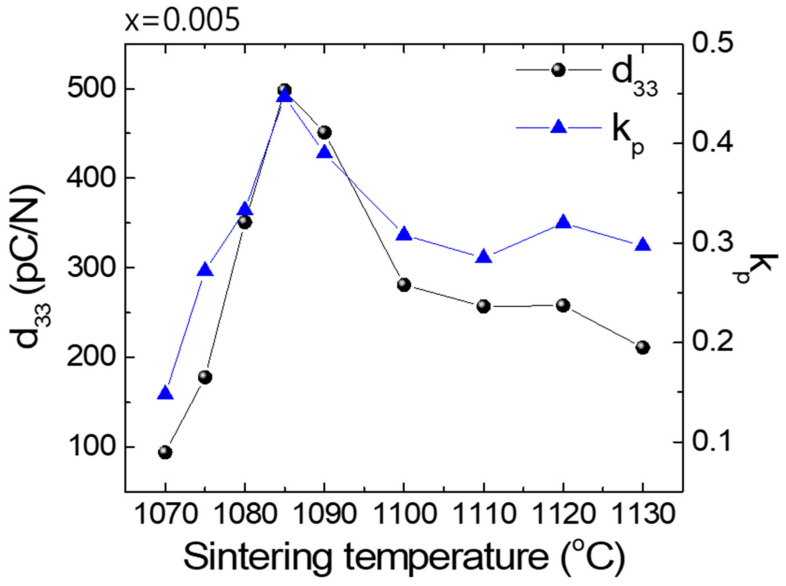
Piezoelectric charge coefficient (d_33_) and electromechanical coupling coefficient (k_p_) of the x = 0.005 specimen sintered at various temperatures for 4 h.

Figure 6 shows the piezoelectric properties of x = 0.005 specimens sintered at various temperatures. Both d_33_ and k_p_ sharply increased as the sintering temperature increased up to 1085 °C but decreased again at higher sintering temperatures. By correlating the results of Figure 2, Figure 5, and Figure 6, we can conclude that the piezoelectric properties were enhanced as the content of large grains increased. The reduction in the piezoelectric properties of the x = 0.005 specimens sintered at temperatures higher than 1085 °C is believed to be attributed to the decrease in T_R-T_ due to the increased vacancy concentration (similar to the explanation in Figure 4) and the decrease in the number of large abnormal grains.

**Figure 7:**
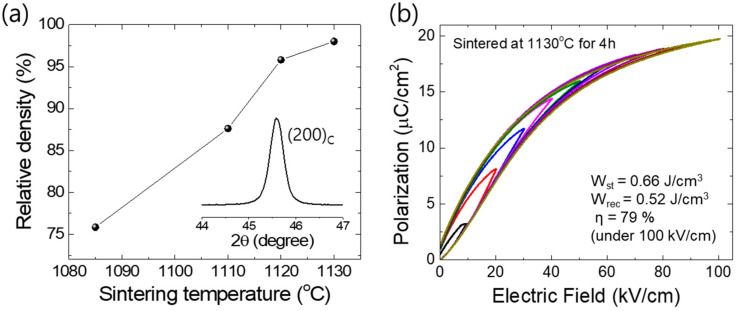
(**a**) Relative density of the x = 0.000 specimen sintered at various temperatures for 4 h. Inset of (**a**) shows the XRD pattern of the (200)_C_ peak of the x = 0.000 specimen sintered at 1120 °C. (**b**) Unipolar P-E curves of the x = 0.000 specimen sintered at 1130 °C for 4 h.

As shown in Figure 2 and Figure 5, the x = 0.000 specimen exhibited a microstructure in which grain growth was suppressed over a wide sintering temperature range from 1070 °C to 1130 °C. However, as the sintering temperature increased, the density of the sintered specimen rapidly increased, and the x = 0.000 specimen sintered at 1130 °C exhibited a high relative density of 98%, as shown in Figure 7a. In addition, owing to the fine grain size of 300–400 nm, the x = 0.000 specimens sintered at high temperatures maintained a pseudo-cubic structure (inset of Figure 7a). Figure 7b shows the unipolar P-E curve of the x = 0.000 specimen sintered at 1130 °C. Owing to the very small grain size and high relative density, a high polarization value was stably obtained without dielectric breakdown, even under a high electric field of 100 kV/cm. 

Furthermore, the weakening of ferroelectricity owing to the fine grains with a pseudo-cubic structure led to a slim P-E curve with very little hysteresis loss. The stored energy density (W_st_), recoverable energy density (W_rec_), and efficiency (η) of the dielectric capacitor are expressed as follows:

(3) $$W_{\mathrm{st}}=\int_{0}^{P_{\max}}\mathrm{EdP}$$

(4) $$W_{\mathrm{rec}}=\int_{P_{r}}^{P_{\max}}\mathrm{EdP}$$

(5) $$\eta \;=\frac{W_{\mathrm{rec}}}{W_{\mathrm{st}}}\times 100\%$$

Due to the high maximum polarization (P_max_) and low remnant polarization (P_r_) of the P-E curve in Figure 7b, the x = 0.000 specimen sintered at 1130 °C exhibited a good energy storage performance of W_st_ = 0.66 J/cm^3^, W_rec_ = 0.52 J/cm^3^, and η = 79%. Therefore, by designing a dense microstructure composed of small grains with a low vacancy concentration, excellent energy storage characteristics can be obtained, even if the piezoelectric characteristics deteriorate.

## 4. Conclusions

In this study, the effects of alkali element deficiency and sintering temperature on the microstructure and electrical properties of 0.96(K_0.46−x_Na_0.54−x_)Nb_0.95_Sb_0.05_O_3_-0.04Bi_0.5_(Na_0.82_K_0.18_)_0.5_ZrO_3_ ceramics were investigated. For the x = 0.000 specimen, grain growth was not observed at any sintering temperature from 1070 °C to 1130 °C because the high ΔG_C_ owing to the very low vacancy concentration was maintained even at high sintering temperatures. In the case of the x = 0.005, 0.010, and 0.015 specimens, the grains satisfying the ΔG ≥ ΔG_C_ condition owing to the increased vacancy concentration grew into abnormal grains by 2D nucleation. The x = 0.005 specimen sintered at 1085 °C exhibited a microstructure composed of large grains of several tens of micrometers and small grains of several micrometers with excellent piezoelectric properties of d_33_ = 498 pC/N and k_p_ = 0.45. For the x = 0.010 and 0.015 specimens sintered at 1085 °C, the piezoelectric properties deteriorated compared to those of the x = 0.005 specimen because the T_R-T_ of x = 0.010 and 0.015 specimens moved to lower temperatures than that of the x = 0.005 specimen. For the x = 0.005 specimen sintered at temperatures higher than 1085 °C, the number of large abnormal grains decreased again as the number of grains satisfying the ΔG ≥ ΔG_C_ condition rapidly increased, owing to the further increased vacancy concentration; accordingly, the values of the piezoelectric coefficients also decreased. The relative density of the x = 0.000 specimen rapidly increased with an increase in the sintering temperature, and the x = 0.000 specimen sintered at 1130 °C with a high relative density of 98% and a fine grain size of 300–400 nm exhibited good energy storage performance of W_st_ = 0.66 J/cm^3^, W_rec_ = 0.52 J/cm^3^, and η = 79%. The results of this study demonstrate that the microstructure of 0.96(K_0.46−x_Na_0.54−x_)Nb_0.95_Sb_0.05_O_3_-0.04Bi_0.5_(Na_0.82_K_0.18_)_0.5_ZrO_3_ ceramics highly depends on the vacancy concentration, and their applications can be diversified depending on the microstructure. These findings are expected to be applied to the study of microstructure control of other KNN-based ceramics.

## Figures and Tables

**Table 1:** Phase fraction of the rhombohedral and tetragonal phases in x = 0.005, 0.010, and 0.015 specimens sintered at 1085 °C for 4 h.

Composition	Phase Fraction (%)	
	Rhombohedral	Tetragonal
x = 0.005	16.74	83.26
x = 0.010	5.79	94.21
x = 0.015	1.65	98.35
